# Multiplex network motifs as building blocks of corporate networks

**DOI:** 10.1007/s41109-018-0094-z

**Published:** 2018-08-29

**Authors:** Frank W. Takes, Walter A. Kosters, Boyd Witte, Eelke M. Heemskerk

**Affiliations:** 1 CORPNET, University of Amsterdam, Nieuwe Achtergracht 166, Amsterdam, 1018 WV The Netherlands; 2 LIACS, Leiden University, Niels Bohrweg 1, Leiden, 2333 CA The Netherlands

**Keywords:** Network motifs, Multiplex networks, Frequent subgraphs, Corporate networks

## Abstract

In *corporate networks*, firms are connected through links of corporate ownership and shared directors, connecting the control over major economic actors in our economies in meaningful and consequential ways. Most research thus far focused on the connectedness of firms as a result of one particular link type, analyzing node-specific metrics or global network-based methods to gain insights in the modelled corporate system.

In this paper, we aim to understand *multiplex* corporate networks with multiple types of connections, specifically investigating the network’s essential building blocks: multiplex *network motifs*. Motifs, which are small subgraph patterns occurring at significantly higher frequencies than in similar random networks, have demonstrated their usefulness in understanding the structure of many types of real-world networks. However, detecting motifs in multiplex networks is nontrivial for two reasons. First of all, there are no out-of-the-box subgraph enumeration algorithms for multiplex networks. Second, existing null models to test network motif significance, are unable to incorporate the interlayer dependencies in the multiplex network. We solve these two issues by introducing a layer encoding algorithm that incorporates the multiplex aspect in the subgraph enumeration phase. In addition, we propose a null model that is able to preserve the interlayer connectedness, while taking into account that one of the link types is actually the result of a projection of an underlying bipartite network.

The experimental section considers the corporate network of Germany, in which tens of thousands of firms are connected through several hundred thousand links. We demonstrate how incorporating the multiplex aspect in motif detection is able to reveal new insights that could not be obtained by studying only one type of relationship. In a general sense, the motifs reflect known corporate governance practices related to the monitoring of investments and the concentration of ownership. A substantial fraction of the discovered motifs is typical for an industrialized country such as Germany, whereas others seem specific for certain economic sectors. Interestingly, we find that motifs involving financial firms are over-represented amongst the larger and more complex motifs. This demonstrates the prominent role of the financial sector in Germany’s largely industry-oriented corporate network.

## Introduction

The field of complex network analysis aims to extract meaningful knowledge from a complex system by analyzing the underlying network structure (Barabási 2016). The obtained insights at the system (or macro) level are the product of interactions between individual entities at the micro level. For example, from friendship relations between individuals at the micro level of a social system, we can observe a small-world structure at the system level (Watts and Strogatz 1998). In case of a contagious disease, by studying interactions between people in the social system, we can understand whether an epidemic is imminent (Pastor-Satorras and Vespignani 2001). In a biological system, the interaction between proteins at the micro level results in a particular biochemical manifestation of the modelled substance (Girvan and Newman 2002). Similarly, in economic networks, an innovation introduced in a particular organization may spread through the organization’s network of contacts (Schweitzer et al. 2009). Indeed, the network approach provides interesting insights in a range of domains, including social, technological and economic systems (Boccaletti et al. 2006).

However, the steps from micro level interaction to macro level insights described above tend to ignore the fact that there is also particular interesting and non-random behavior at the intermediate *meso* level. In this perspective, smaller groups of nodes, connected in a particular way, play a crucial role in the functioning of the modeled system. Although the somewhat loose description of meso level patterns above may for example also include variable size communities (Fortunato 2010), here we refer to a more precise network pattern, namely that of network motifs (Alon 2007; Märtens et al. 2017; Milo et al. 2002; Ohnishi et al. 2010; Paranjape et al. 2017; Romijn et al. 2015; Wernicke 2005; Zhang et al. 2014). A *network motif* is a pattern consisting of a relatively small number of nodes and connections, appearing in the same configuration at frequencies much higher than what we would expect in a similar random network.

For social networks in general, the systematic analysis of motifs introduces a novel perspective in a long standing debate in the social sciences on the relation between micro and macro level properties of social systems (Coleman 1998). Motifs have furthermore been proven instrumental in a number of systems with a clear network perspective, for example explaining the function of neuron groups in brain networks (Märtens et al. 2017) and the formation of particular group structures in social networks (Benson et al. 2016). Consequently, network motifs are frequently considered to be the higher order *building blocks* of complex networks (Milo et al. 2002; Benson et al. 2016).

In a real-world setting, a complex network may have multiple types of interaction going on between its individual entities. This observation is methodologically accommodated by so-called *multiplex networks* (Dickison et al. 2016; Gomez et al. 2013; Kivelä et al. 2014; Cardillo et al. 2013) (or edge-colored networks, see the “Multiplex networks and motifs” section) in which there may be multiple “layers” at which network interaction is taking place. For example, in a real-life social network, there may exist both friendship and co-worker relationships, and the two may overlap at times. In an economic network, the diffusion of an innovation may occur through both supplier relationships and employee movement. Although both the multiplex aspect as well as the study of network motifs are commonly undertaken tasks in network analysis, the combination of the two, i.e., *detecting multiplex network motifs*, is to the best of our knowledge an underaddressed problem. Indeed, particular combinations of links at different levels of the network may define how the network as a whole grows, operates and functions; the essential building blocks of a network may very well be based on multiple types of interaction. This paper extends our previously introduced algorithmic framework for multiplex motif detection (Takes et al. 2017), focusing in detail on the meaning and consequences of these motifs in corporate networks.

*Corporate networks*, in which governance and power-related connections between corporations are the object of study, play a crucial role in understanding our global corporate system (Vitali et al. 2011a; Carroll 2013). They have been proven instrumental in for example explaining how firms exert power, coordinate their behaviour and regulate competition (Davis et al. 2003; Windolf 2002). A node in a corporate network represents a firm or a corporation, whereas a link may denote different types of relationships, such as trade (Wilhite 2001), loans (Battiston et al. 2016) and supplier relationships (Choi and Wu 2009). In this paper we focus on two different types of links in corporate networks that pertain to corporate control, namely ownership and board interlocks.

**Fig. 1:**
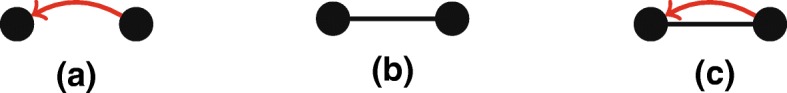
Link types in the multiplex corporate network. **a** Ownership link, **b** Board interlock link, **c** Multiplex link

Firstly, an *ownership* relation indicates that a particular firm owns part of another firm, in the remainder of this paper indicated by a directed link as shown in Fig. 1a. This type of connection between firms has been shown to be extremely important in understanding relations of corporate control and value flow (Vitali et al. 2011b). Network analysis of ownership ties has been able reveal patterns related to corporate tax evasion (Richardson et al. 2016). Ownership links have furthermore proven instrumental in identifying offshore financial centers (Garcia-Bernardo et al. 2017). The second type of a link, a *board interlock*, represents the fact that firms often have so-called interlocking directorates, meaning that a director is a member of the board of two organizations, which we represent using an undirected link as shown in Fig. 1b. This type of link has been shown to play a key role in for example the understanding of innovation diffusion (Davis 1991), information exchange and transnational business elite cohesion (Heemskerk and Takes 2016). In fact, a long line of research in the social sciences has dealt with the particular causes and consequences of these interlocking directorate networks (Mizruchi 1996).

Crucially, the abovementioned two types of links often occur together (depicted using the multiplex link in Fig. 1c), as both ownership and board interlocks are instruments by which one firm can influence or exert power over the other. Indeed, also in our data, these two types of links often coincide, at numbers that are thousands of times higher than one would expect (see the “Corporate network data” section), calling for a multiplex network approach.

Many existing methods common in complex network analysis have been applied to the aforementioned corporate networks in order to better understand their structure, dynamics and function. Simple metrics such as density, average degree and average clustering coefficient proved crucial in assessing the cohesiveness of corporations across countries (Kogut 2012; van Veen and Kratzer 2011). Centrality measures were applied to assess the powerful and well-connected firms within countries, and on a more global level the power of particular countries (Takes and Heemskerk 2016). Community detection has been used to understand the formation of global business groups and to shed light on debates regarding the formation of a transnational business elite (Heemskerk and Takes 2016).

In this paper we for the first time set out to explore the meso level of these corporate networks, dealing with the topic of motif detection in the multiplex network of ownership and interlocking directorates. We choose to focus on the corporate network of the largely industrial country of Germany, for which previous studies have shown that data quality in terms of completeness is sufficiently high (Garcia-Bernardo and Takes 2017). This paper provides three contributions. First, in order to perform multiplex motif detection, we modify and extend existing algorithms for motif detection in networks with homogenic links. In particular, we modify the subgraph enumeration step, so that it can exhaustively enumerate multiplex subgraph patterns. In addition, we introduce a layer encoding scheme that then enables the deterministic counting of multiplex subgraphs. The second contribution results from the fact that layers of a multiplex network are not independent, which requires a new null model that takes into account the relatedness of different link types, by also explicitly modelling the co-occurrence of link types. These two contributions together provide a methodological advancement in network motif detection, as the methodology explained in the “Approach” section, which in general builds on the framework which we proposed in Takes et al. (2017), can be applied to any multiplex network. Third, our experiments on the German corporate network data result in a number of interesting findings typical for the German economy and explanatory for the corporate governance practices in several of the country’s economic sectors.

The rest of this paper is organized as follows. After discussing related work in the “Related work” section, we turn to the formal definitions of network patterns and motifs as well as relevant evaluation metrics in the “Preliminaries” section. The “Approach” section describes the new multiplex approach to subgraph enumeration as well as the adjusted null model. Then, using the corporate network data described in the “Corporate network data” section, we perform experiments in the “Experiments” section. Finally, the “Conclusion” section provides concluding remarks and suggestions for future work.

## Related work

In this section we discuss related work on motif recognition, corporate networks and the analysis of multiplex networks.

Motif recognition has been applied in a number of network types, including social networks (Benson et al. 2016), biological networks (Milo et al. 2002) and brain networks (Märtens et al. 2017). The problem of motif recognition, of which the major step is subgraph enumeration, is interesting from a computational point of view, as enumerating subgraph isomorphism is an NP-complete problem (Romijn et al. 2015; Kuramochi and Karypis 2005), and each subset of nodes in a graph has to be compared against all known (possibly isomorphic) subgraphs. Thus, for larger graphs and larger subgraph sizes, exhaustive enumeration is prohibitive. Therefore typical motif recognition algorithms either run on very small inputs, or instead of an exhaustive list, provide merely an approximation of the frequency of the network motifs (Romijn et al. 2015). One way to address this is to only study part of the network’s subgraphs, requiring input on either which particular subgraphs should be counted or what threshold the frequency of the motif should pass (Ghazizadeh and Chawathe 2002). Other motif recognition algorithms avoid the computational limitations by only finding a specific subset of patterns, or discover only patterns with certain topological characteristics, such as dense subgraphs (Haiyan et al. 2005). Methods like G-TRIES (Ribeiro and Silva 2010), FANMOD (Wernicke 2005), and SUBENUM (Saeed and Saeed 2015) find only induced subgraphs, the type of subgraphs that we also consider in this paper.

Recently, a new trend in motif recognition is that in order to avoid the computational difficulties of motif enumeration, the focus becomes that of motif countings (Paranjape et al. 2017; Benson et al. 2016). Without explicitly enumerating them, the goal is to obtain exact counts for motifs of a particular shape and/or size. The advantage is that these counting methods are significantly faster. Yet the disadvantages are that currently they do not work for motifs larger than three nodes, nor do they have the option to assess which nodes are involved in which motifs. Because ultimately we are interested in the composition of motifs found in corporate networks and what insights they provide, motif enumeration (and not counting) is the specific focus of this paper.

Although corporate networks have extensively been analyzed in terms of network topology (Vitali et al. 2011b), centrality (Takes and Heemskerk 2016) and community detection (Heemskerk and Takes 2016), few papers deal with detecting motifs in corporate networks. In Ohnishi et al. (2010), interfirm relationships based on materials and services exchanged are investigated up to size three, counting V-shaped and triangle-shaped network structures, essentially limiting the study to one-layer motifs of size three. Within the field of board interlock research some studies have attempted to look at pre-defined well-known motif like patterns such as star and pyramid configurations (Windolf and Beyer 1996) and subsequently counting their occurrence in networks of interlocks (Heinze 2004) or studying the sequences of such patterns over time (Stark and Vedres 2006). However, as far as the authors of this work are aware, there are no studies of multiplex motifs in corporate networks based on board interlock and ownership relations, as considered in this paper.

Real-world multiplex networks (such as our corporate networks) in which multiple types of interaction simultaneously take place, have extensively been studied and classified. An excellent overview can be found in Kivelä et al. (2014). Important to note is that here we focus on networks in which the same set of nodes is connected by different (possibly multiple) types of relationships. These networks are sometimes also called multi-relational, multi-dimensional or multi-layer networks. A good overview of these naming conventions and accompanying definitions can be found in Boccaletti et al. (2014). Importantly, the goal is to not lose information by aggregating the different link types of the network, and to take advantage of the insights that result from the multiple types of interaction (Dickison et al. 2016). In this light, a number of network characteristics and methods have been devised, including centrality (Solé-Ribalta et al. 2014) and community detection (Mucha et al. 2010). This work aims to contribute to the broader field of multiplex network analysis by means of a new method of analysis at the meso level: the discovery of multiplex network motifs.

## Preliminaries

Before we can formulate our exact problem statement in the “Motif detection problem” section, this section introduces elementary network concepts, first in the “Networks and motifs” section for simple (directed) networks and then for multiplex networks in the “Multiplex networks and motifs” section. The “Motif evaluation metrics” section discusses how the obtained patterns can be evaluated quantitatively. Here, we build on the framework which we previously introduced in Takes et al. (2017).

### Networks and motifs

A *graph* or *network* *G*=(*V*,*E*), consists of a finite set of nodes *V*=*V*(*G*) (also called objects or vertices) and a set of directed edges *E*=*E*(*G*)⊆*V*×*V* (also called relationships or links). Nodes are identified using some unique identifier (ID) or label. We assume that there are no parallel edges or self-loops. A graph *g* is a *subgraph* of graph *G* if and only if *E*(*g*)⊆*E*(*G*) and *V*(*g*)⊆*V*(*G*), where all nodes incident with an edge in *E*(*g*) occur in *V*(*g*). A subgraph *g* is an *induced subgraph* of *G* if for any pair of nodes *u*,*v*∈*V*(*g*), it holds that if (*u*,*v*)∈*E*(*G*) then (*u*,*v*)∈*E*(*g*). We only consider *connected induced subgraphs* in which all nodes are (indirectly) linked through edges, ignoring link direction. The size *k* of a subgraph *g* is its node count, i.e., *k*=|*V*(*g*)|.

The *pattern* of a (sub)graph is its abstract representation without particular identifiers or labels. All isomorphic (sub)graphs thus have the same pattern. Let *I* denote the collection of all patterns. We define *S*^*i*^(*G*) as the set of subgraphs of pattern *i*∈*I* in graph *G*. The *frequency* of pattern *i*∈*I*, denoted |*S*^*i*^(*G*)|, is the number of occurrences of pattern *i* in graph *G*. A *motif* is a pattern that is considered significant according to a particular frequency-based comparison or metric (as further discussed in the “Null model” section). The set of all motifs of size *k* in graph *G* is denoted *M*_*k*_(*G*), and the set of all motifs *M*(*G*)=∪_*k*_ *M*_*k*_(*G*).

### Multiplex networks and motifs

A *multiplex graph* (or *network*), denoted $\mathcal {G} = (V, E, J)$, is a graph that contains multiple types of edges. The collection of edge types is called *J*. We use $E_{j}(\mathcal {G})$ with *j*∈*J* to refer to the set of edges of type *j*. There is at most one edge of a certain type in the same direction between any two nodes, meaning that if there are multiple edges between two nodes, they are of different types. An alternative definition of this data structure would be that of an *edge-colored* graph, in which each edge has an associated color corresponding to its edge type or combination of types. In a *multiplex induced subgraphg* it holds that for any pair of nodes *u*,*v*∈*V*(*g*) in subgraph *g* and for each type of edge *j*∈*J* that if $(u,v) \in E_{j}(\mathcal {G})$ then (*u*,*v*)∈*E*_*j*_(*g*). Following similar definitions for patterns and frequency as in the “Networks and motifs” section (e.g., introducing $S^{i}(\mathcal {G})$), a *multiplex motif* is a multiplex pattern that is considered significant according to a particular frequency-based comparison (as further discussed in the “Null model” section). The set of all motifs of size *k* in multiplex graph $\mathcal {G}$ is denoted $\mathcal {M}_{k}({\mathcal {G}})$, and the set of all multiplex motifs ${\mathcal {M}({\mathcal {G}})= \cup _{k}\, \mathcal {M}_{k}({\mathcal {G}})}$.

### Motif evaluation metrics

The significance of a pattern (counted subgraph) and classification as a motif is the final step in the process from subgraph counts to motifs. Given the large number of different subgraphs that may exist, qualitative evaluation of the obtained patterns is difficult. Below we describe two quantitative methods to determine pattern significance, essentially defining a function *f* which takes as input both a pattern and the network in which that pattern was found. It then outputs a numeric value indicating the significance of the motif. The first function is based solely on the empirical graph and patterns of a similar size, whereas the second performs a comparison with a null model of the network:

- The *concentration*$c(i,\mathcal {G})$ of a pattern *i* (of size |*i*|) in graph $\mathcal {G}$ is the ratio between its frequency and the frequencies of all patterns of the same size (see Wernicke (2005)), expressed as a percentage:
  $$c(i,\mathcal{G}) = \frac{\left|{S}^{i}(\mathcal{G})\right|}{\sum_{j \in I, |j|=|i|}\left|{S}^{j}(\mathcal{G})\right|} \ \cdot \ 100\% $$
- The *ratio*$R(i,\mathcal {G})$ of a pattern *i* in graph $\mathcal {G}$ given a set of random multiplex graphs *Y* (the null model), is defined as follows:
  $$r(i,\mathcal{G}) = \left|{S}^{i}(\mathcal{G})\right| \cdot \left(\frac{\sum_{\mathcal{H} \in Y} \left|{S}^{i}(\mathcal{H})\right|}{|Y|}\right)^{-1} $$
  When the ratio is larger than 1, the probability of pattern *i* appearing in the empirical network is larger than the probability of *i* appearing in a random graph (Wernicke 2005). Various random graph models may be used as a null model, and a suitable multiplex model should be used that takes into account the interdependencies between the different link typess (see the “Corporate network data” section). Such a null model is proposed in the “Null model” section.

Given the full set of subgraphs of a network, we may define a cut-off value or choose to study only the motifs ranked highest according to the metrics defined above.

### Motif detection problem

Now that we have the basic definitions of multiplex networks, subgraphs, patterns and motifs, we can come to the main problem statement which we set out to address in this paper:

Given as input a multiplex graph $\mathcal {G}$, motif size *k* and significance evaluation function *f*, determine the set of multiplex motifs $\mathcal {M}_{k}({\mathcal {G}})$.

This problem consists of three subproblems:

1. Enumerating all multiplex subgraphs (adressed in the “Multiplex SubEnum” section).
2. Counting the frequency of each multiplex subgraph (addressed in the “Multiplex subgraph counting” section).
3. Motif significance testing, applying the metrics from the “Motif evaluation metrics” section. For the “ratio” metric, this requires a suitable multiplex null model (addressed in the “Null model” section).

## Corporate network data

This section first describes the raw data as well as the method of network construction in the “Network construction” section, after which the “Network characteristics” section provides elementary properties and characteristics of the resulting multiplex network.

### Network construction

The corporate network data considered in this work was gathered from the Orbis database (http://orbis.bvdinfo.com) by Bureau van Dijk. Orbis is a common and frequently used corporate database, sourced from official country registrars such as chambers of commerce as well as other collection agencies. Orbis is often labeled as one of the most reliable and complete sources of corporate data (Vitali et al. 2011b; Heemskerk and Takes 2016; Garcia-Bernardo and Takes 2017). For our corporate network, we extracted all active German companies for which ownership and/or board information was available in November 2015. We also extracted identifiers of the firm’s economic sector, resulting in the list of sectors shown in the leftmost column of Table 1. Consequently, the set of nodes of our network consists of exactly these firms, where each node has one associated economic sector attribute.

**Table 1:** Division of firms over economic sectors

Sector	Ownership		Board interlock		Multiplex	
Bank	474	1.25%	865	1.41%	972	1.29%
Financial	4 648	12.32%	6 250	10.21%	8 338	11.08%
Foundation/research	55	0.14%	51	0.08%	88	0.12%
Industrial	32 350	85.75%	53 767	87.84%	65 484	87.05%
Insurance	19	0.05%	26	0.04%	34	0.05%
Mutual & pension fund	112	0.30%	175	0.29%	213	0.28%
Private equity	29	0.08%	30	0.05%	37	0.05%
Public authority	22	0.06%	31	0.05%	41	0.05%
Venture capital	15	0.04%	14	0.02%	17	0.02%

In addition to the node-specific data specified above, we extracted all significant ownership relations between these firms with a share of at least 5%, a common threshold at which a stake is considered significant. It should however be noted that the majority of ownership links is in fact greater than 50%, and that this weight is not taken into account in the remainder of this paper. Together, these links form a directed network *G*_*a*_ in which a link (*u*,*v*)∈*E*_*a*_ indicates that firm *u* owns a part of firm *v* and is thus able to exert control over it. We also extracted for all firms their top executives (chief officers and directors) and supervisory board. The creates a bipartite network that connects firms to directors if the director serves at the board of that firm. This bipartite network can be projected onto an undirected one-mode network *G*_*b*_ in which links {*u*,*v*}∈*E*_*b*_ indicate that *u* and *v* share at least one director. We now have a multiplex network $\mathcal {G}$ with a layer of directed ownership links *E*_*a*_ and a layer of undirected board interlock links *E*_*b*_.

It should be noted that in this paper we observe the structure of this multiplex corporate network at one point in time. Obviously, not every link appeared at the same time, and during the evolution of this network up to the day of the snapshot, changes may have occurred to the structure of the network. It should be noted that because our data is not timestamped, we do not explicitly model these types of dynamic changes. Rather, we focus on the current structure of the multiplex corporate network of Germany, and the interesting characteristics (see the “Network characteristics” section) and patterns (see the “Experiments” section) that we can derive from this network.

### Network characteristics

Table 2 reports basic network statistics of our corporate network dataset such as the number of nodes, links, density, and average local clustering coefficient. See for example (Barabási 2016) for definitions of these elementary network metrics. The average local clustering coefficient was computed by ignoring link direction, averaging over all nodes the value of the local node clustering coefficient, which in turn was computed as the fraction of closed triangles amongst a node’s direct neighbors. For each link type in the multiplex network we report the number of nodes with a degree greater than one. Alternatively one could state that the network has 75 224 nodes and that the column “Nodes” of Table 2 indicates how many nodes have a degree that is not equal to zero. As the motif detection algorithms considered in the “Approach” section model an undirected network as a symmetric directed network, the board interlock links reported in Table 2 are fully symmetric.

**Table 2:** Network statistics

Network	Nodes	Links	Density	Clustering
Ownership	37 724	31 506	2.25·10^−5^	0.033
Board interlock	61 209	175 108	4.67·10^−5^	0.384
Multiplex	75 224	195 073	1.72·10^−5^	0.277

**Fig. 2:**
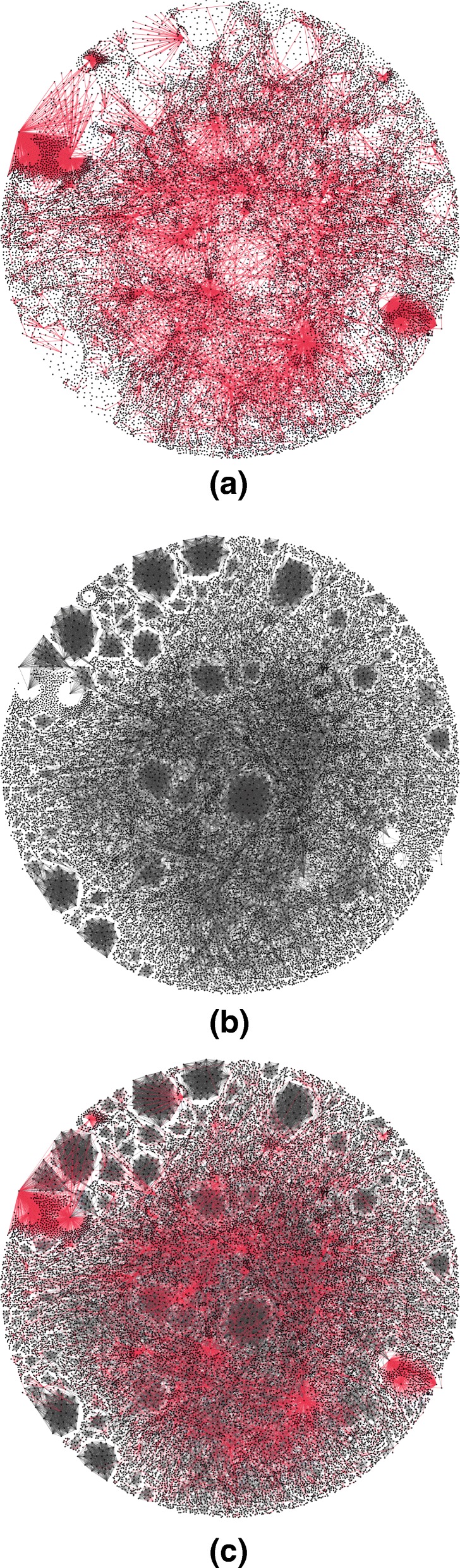
Network visualizations. Created using ForceAtlas2 (Jacomy et al. 2014) in Gephi (http://gephi.org) using the “stronger gravity” option. **a** Ownership network, **b** Board interlock network, **c** Multiplex network

Three visualizations are given in Fig. 2, showing the ownership links in Fig. 2a and the board interlocks in Fig. 2b. The combination of the two layers is shown in Fig. 2c. Note that the placement of nodes is different in each figure. From the visualizations we can already see that there is significant overlap in the different layers of the multiplex network. Indeed, 23 709 nodes (32% of the nodes in the multiplex network) are involved in both ownership and board interlock links. Similarly, 11 541 edges are what we call multiplex links: connecting nodes with both an ownership link and a board interlock, constituting 37% of the ownership links, 6.7*%* of the board interlocks and in total 5.9*%* of the multiplex network. To understand the significance of the link overlap, the empirical link counts can be compared to a multiplex network with randomly generated layers.

In a directed network with *n* nodes, there are *n*(*n*−1) potential links. If there are *m* links actually present, between a randomly chosen node pair, a link has a *m*/*n*(*n*−1) probability of being present. So for the ownership network with 75 224 nodes and 31 506 links, a randomly chosen directed link only has a 0.0005568*%* chance of occurring. However, with 175 108 undirected links in the board interlock network, in the empirical data no less than 23 709 node pairs share at least one ownership link and a board interlock link.

## Approach

This section first explains how the enumeration and counting steps of an existing state-of-the-art subgraph enumeration algorithm can be adjusted to handle multiplex network data in the “Multiplex subgraph enumeration and counting” section. Next, the “Null model” section describes a null model that is suitable for multiplex networks. Note that this approach builds on the methodology which we previously introduced in Takes et al. (2017).

### Multiplex subgraph enumeration and counting

As discussed in the “Related work” section, a number of efficient subgraph enumeration algorithms have been devised for simple one-layer networks. Below we first briefly discuss the SUBENUM (Saeed and Saeed 2015) algorithm for subgraph enumeration on which our approach is based, before introducing necessary algorithmic adjustments for multiplex networks.

#### SUBENUM

**Fig. 3:**
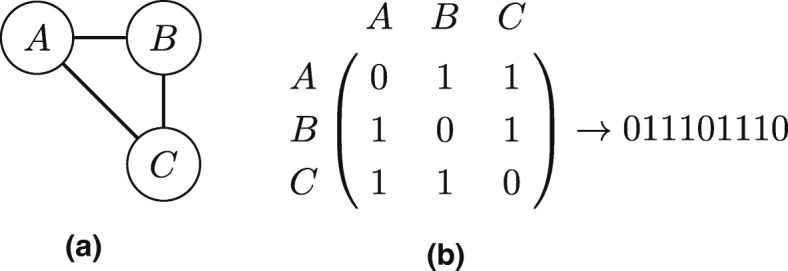
Pattern labeling. **a** Undirected graph, **b** Label of this graph

The input of SUBENUM is a directed network. The output is a set of subgraphs and their frequencies. At the basis is the Enumerate Subgraph algorithm (ESU) (Wernicke 2005), which counts subgraphs in directed unweighted graphs. It loops over all nodes starting at the node with the lowest ID, recursively expanding on every neighboring node with a higher ID until the set of nodes is of size *k*. The resulting set of nodes including the edges that exist between these nodes, is an induced subgraph, which is then given a canonical label with the NAUTY (McKay and Piperno 2014) algorithm. This label is guaranteed to be equal for all isomorphic subgraphs. Undirected edges are represented as symmetric directed links. See Fig. 3 for a simple example of this labeling step, applied to a toy size undirected network. SUBENUM (Saeed and Saeed 2015) is a parallel variant of the ESU algorithm, solving thread load balancing issues by performing the aforementioned expanding process on the edges instead of nodes. To work round memory limitations, it adjusts the way subgraphs are checked for isomorphism, using a two phase isomorphism check where intermediate results are stored to disk. As these changes mostly relate to the precise implementation and not the algorithmic concepts, we refer the reader to Saeed and Saeed (2015) for a more detailed description of these practical aspects.

#### Multiplex SUBENUM

**Fig. 4:**
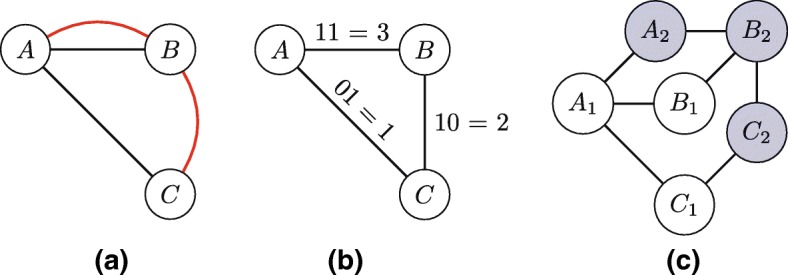
Multiplex subgraph encoding: from multiplex links to binary labeled (weighted) edges to colored nodes. **a** Multiplex links, **b** From binary labels to weighted edges, **c** Colored nodes

The proposed multiplex adaptation is a two-step process: adjusting the subgraph recognition algorithm SUBENUM (discussed below) and adjusting the isomorphism detector NAUTY accordingly (discussed in the “Multiplex subgraph counting” section). Here we exploit the fact that any multiplex graph $\mathcal {G}$ can be expressed as a directed labeled graph *G*^′^. Instead of explicitly storing multiple edge types, the multiplex graph is converted into a directed labeled graph in which each edge has a label based on the edge types present between the two nodes that it connects. The label consists of a binary string of length *J* (the number of layers/link types), of which the bit at index *i* is equal to 1 if an edge of type *i* is present and 0 otherwise (note an ordering is applied to *J* so an index can be assigned to each edge type). This binary label can be seen as an edge weight, as illustrated in Fig. 4a and b. It should be noted that this conversion to a seemingly weighted graph *G*^′^ does not imply that we are suddenly dealing with a weighted graph; we are merely encoding layer presence or absence, and summarize this with a number.

**Fig. 5:**
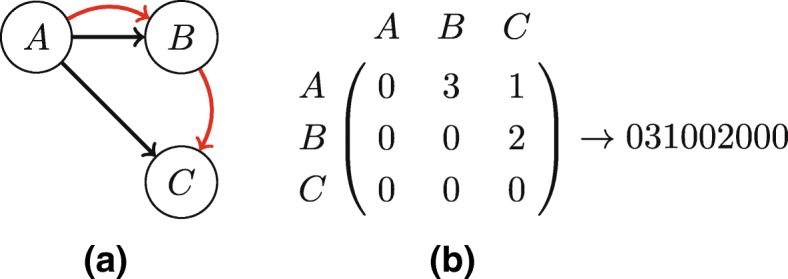
Pattern labeling in directed multiplex networks. **a** Directed multiplex graph, **b** Labels of this graph

Although we now have weights/labels representing the layers, the original ESU algorithm does not handle labels nor weights. Therefore we propose that when the algorithm encounters a subgraph, a label is created based on the adjacency matrix with weights representing the layer encoding, as shown in Fig. 5. Then, to adapt SUBENUM to handle weighted graphs, we only have to adapt the label constructor so that it incorporates the edge weights.

#### Multiplex subgraph counting

The second step is adjusting NAUTY to handle the weighted graphs, for which we use node-colored graphs, which have multiple node types (colors). This method is similar to the suggestion for expressing weighted graphs given in NAUTY’s documentation (McKay and Piperno 2014). We create a new node-colored graph graph *G*^″^ from *G*^′^, which is the graph with binary labels representing multiplex graph $\mathcal {G}$ as discussed above. The number of node colors is equal to |*J*|, and each color is used to express a single edge type, according to the binary label. For each node in *V*(*G*^′^), a set of |*J*| colored nodes is created in *V*(*G*^″^). So for every node *A*∈*V*(*G*^′^), a set {*A*_1_,*A*_2_,…,*A*_|*J*|_} with different colors is added to *V*(*G*^″^). Then, for 1≤*j*<|*J*|, every *A*_*j*_∈*V*(*G*^″^) is connected to *A*_*j*+1_ by adding an undirected edge (*A*_*j*_,*A*_*j*+1_) to *E*(*G*^″^). This creates a string of colored nodes for each node in the original network. Then, crucially, an edge between two nodes *A*_*j*_ and *B*_*j*_ is used to express the presence of the *j*^*th*^ edge type encoded in the binary label. An example with undirected edges can be seen in Fig. 4c, where the multiplex graph from Fig. 4a with two types of edges is shown rewritten with two types of colored nodes.

### Null model

Random graph models exist in many flavors, and include for example the Erdős-Rényi model (Erdős 1959), the Chung-Lu model (Chung and Lu 2002), the Park-Newman model (Newman and Park 2003), and the stub-matching model (Bender and Canfield 1978). Each of these models preserves a different type of network property, such as the average degree, the degree distribution or the precise degree sequence. In our case, the null model is employed to understand the significance of motifs, which are essentially higher order network patterns. Therefore, we wish to preserve the lower order properties, i.e., the precise degrees of the nodes in the network. In addition, the null model should handle the strong dependencies between the different layers. As we noted in the “Corporate network data” section, 5.9*%* of all edges overlap, e.g., there is both an ownership link and a board interlock. The quick calculation presented at the end of the “Network characteristics” section reveals that as a result of the low density of the networks of each link type, merging two separately generated random networks will have far too few overlapping edges. Indeed, the concept of interlayer assortativity (Dickison et al. 2016), sometimes (although in a slightly different context) also called interlayer dependency, coupling or interconnectedness (Radicchi and Arenas 2013), is common across different multiplex networks and has to be preserved in the null model.

Given the considerations above, we build on the stub-matching model (Bender and Canfield 1978), which generates random networks with a particular fixed in and outdegree sequence, by definition also preserving the exact number of nodes. Furthermore, to ensure that degree sequences are fixed for all edge types, each combination of edge types is modeled separately, fixing the node degrees for each (combination of) link type(s). Thus, we model in total 2^|*J*|^−1 different networks (recall that *J* is the set of link types). This is a mere three network models in our case, namely for the ownership links, the board interlocks and the combined “multiplex link”.

In our particular case, a second challenge is the fact that the board interlock network is a product of the projection of the bipartite network linking firms and directors to a firm-by-firm network which links firms based on shared directors. As such, a relatively large number of cliques exists in the empirical network, effectively resulting from directors with three or more positions. Not explicitly modelling this phenomenon would simply result in all discovered motifs being clique-like. So to ensure that this particular aspect is preserved, the undirected interlock network is modeled at the bipartite level. For this, we again employ the stub-matching model (Bender and Canfield 1978). We encode the node type (firm or director) by enforcing that directors only have a particular outdegree value, and firms only a particular indegree value. The subsequent conversion to an undirected network is trivial, after which a regular projection to the one-mode firm-by-firm network can be made. It should be noted that in our case, the same bipartite projection step should be done for multiplex links, because part of a multiplex link is an interlock edge. Finally, the different networks for each of the 2^|*J*|^−1 link type combinations (three in our case) are combined into one multiplex network.

Ultimately, the use of this multiplex model allows us to generate a set *Y* of networks to which the empirical network data can be compared using one of the evaluation functions presented in the “Motif evaluation metrics” section.

## Experiments

This section describes our experimental setup in the “Experimental setup” section, followed by a description of the results for motifs of particular sizes in the “Motif results” section and the corporate network as a whole in the “Discussion” section.

### Experimental setup

The multiplex motif detection approach explained in the “Approach” section will be applied to the corporate network dataset from the “Corporate network data” section. The null model that serves as a baseline for assessing the significance of obtained results (see the “Null model” section) is generated using 1 000 samples, as suggested in Wernicke (2005). As for the evaluation metrics proposed in the “Motif evaluation metrics” section, we manually set a cut-off value of 5 for the ratio and 0.01*%* for concentration. This means that a discovered subgraph becomes significant, i.e., a motif, when compared to random graphs with the same degree sequence, it is 5 times more frequent and makes up more than 0.01*%* of all the patterns of the same size. Addressing the problem statement posed in the “Motif detection problem” section, we run the full motif detection pipeline for *k*=3, *k*=4 and *k*=5. To keep running time within reasonable limits, we run the algorithm up to motif size *k*=5. Further experiments on the running time and memory usage are beyond the scope of this work, as only constants and not orders are added to the subgraph enumeration algorithm on which the method is based. An implementation of the approach can be found at the supplementary material website http://liacs.leidenuniv.nl/~takesfw/multiplexmotifs.

**Fig. 6:**
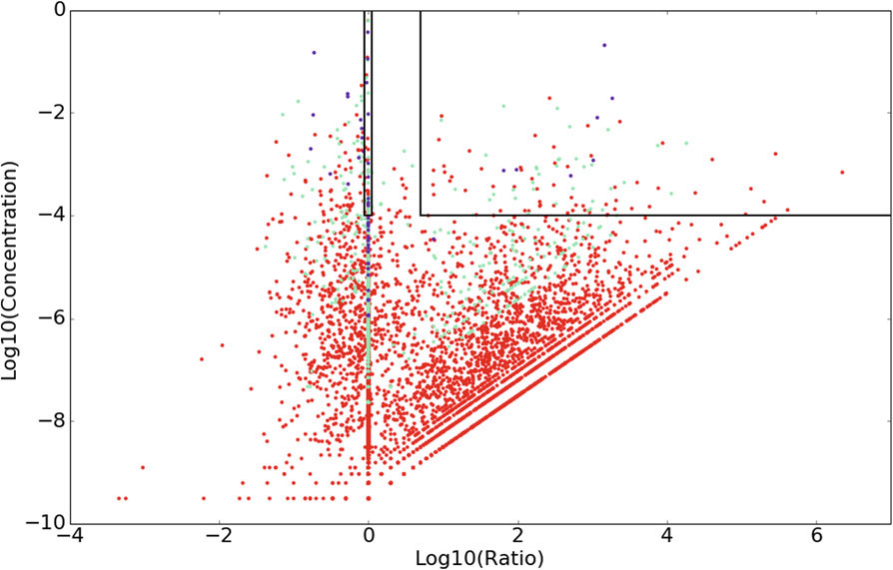
Ratio (horizontal axis) vs concentration (vertical axis) for all patterns. Top right box indicates cut-off values. Patterns of size 3 in blue, size 4 in green and size 5 in red

Figure 6 (note the asymmetric logarithmic axes) shows how the chosen cut-off values capture the truly interesting ensure that only a small part of the discovered subgraphs are labeled significant and thus become motifs. The results of this selection are shown in Table 3, listing for increasing values of *k* the number of discovered patterns (enumerated subgraphs) and motifs (significant patterns). The chosen cut-off values of concentration and ratio reduce the 23 048 patterns to only 135 motifs. Note how, even for smaller subgraph sizes, not all possible subgraphs are present in the empirical network. The only exception are the undirected board interlocks of size 3 (two patterns; a triangle and a wedge) and size 4 (six patterns).

**Table 3:** Number of discovered patterns and motifs per network

	Pattern size				Motif size			
	3	4	5	All	3	4	5	All
Ownership	11	63	391	465	3	4	6	13
Board interlock	2	6	21	29	0	2	10	12
Multiplex	58	1 132	21 858	23 048	14	48	73	135

As the motif size increases, more complex motifs are found, for which it is not always trivial to understand the composition. To address this, *node-specific attributes* can be used to characterize the discovered motifs. We can then define for each motif the extent to which this motif contains nodes with a certain attribute value. To better understand and still capture interesting aspects of these motifs, we can use the economic sector of a node (an overview of this attribute shown in Table 1). Then for a motif, we can look at all subgraphs in the empirical data that make up this motif, and determine for each economic sector the percentage at which it is involved in that motif. A simple baseline is to say that in a random graph, the distribution of economic sectors over a particular subgraph pattern should on average be equal to that of the entire graph. If a certain motif exhibits substantially more nodes of a certain sector, then this may suggest that the considered motif is characteristic for that particular economic sector. We will highlight motifs with such an interesting sector composition throughout this section.

As the motifs we identify strongly relate to real-world patterns in corporate control, they immediately suggest interpretations. These suggested interpretations are of course subject to further investigation, given that the data is not timestamped (as noted in the “Corporate network data” section). In particular, we can only assess the static existence of particular relationships and motif occurrences, but no direct causal relationships related to the order in which links appeared. The discovered motifs however do allow us to see the value of our approach for the domain of interlocking directorates and corporate governance research (Mizruchi 1996; Kogut 2012). Throughout this section we will demonstrate the use of this exciting new method of multiplex motif detection to dissect corporate networks and to understand the small microstructures that play a role in their structural composition.

### Motif results

For the discovered motifs of each size, in this sector we discuss their generic composition, as well a few with exceptionally high concentration, ratio or an interesting economic sector composition. An exhaustive list of the motifs can be found at the supporting website http://liacs.leidenuniv.nl/~takesfw/multiplexmotifs.

**Fig. 7:**
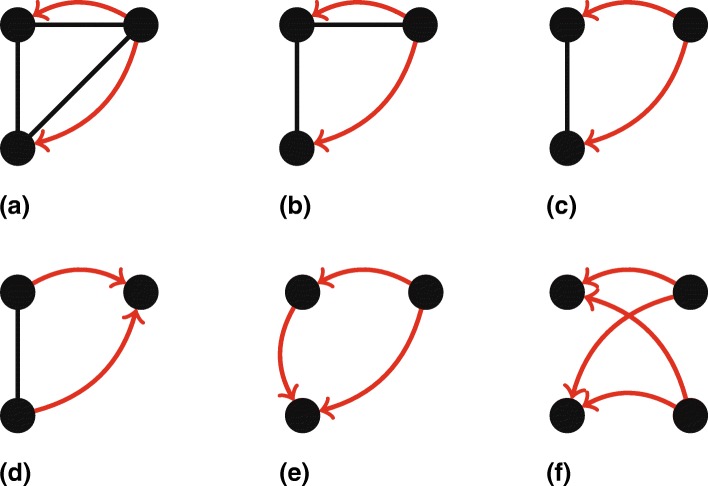
Highlighted motifs of size 3 and 4. Values of *r* and *c* denote respectively ratio and concentration, cf. the “Motif evaluation metrics” section. **a** Motif of size 3. *r*=*∞*,*c*=0.945*%*, **b** Motif of size 3. *r*=1032, *c*=0.118*%*, **c** Motif of size 3. *r* = 1170, *c* = 0.805*%*, **d** Motif of size 3. *r* = 516, *c*=0.059*%*, **e** Motif of size 3. *r*=278, *c*=0.418*%*, **f** Motif of size 3. *r*=2024, *c*=0351*%*

**Size 3.** A total of 8 out of the 14 multiplex motifs of size 3 features a multiplex link, showing how apparently investments frequently go together with shared directorships. This observation is in line with previous work, where these multiplex ties are considered relevant to exercise additional control of the investment and increase the de facto concentration of ownership (Mizruchi 1996). The highlighted motifs in Fig. 7 as such present an original insight in how investors use board interlocks in combination with their shareholdings. Note that although similar in shape, Fig. 7a–b are in fact distinct subgraph patterns, as we always look for induced subgraphs.

The frequent occurrence of the motif in Fig. 7a for instance is in line with the practice where an investor sits on the board of the firms it invests in. Furthermore, Fig. 7b suggests a situation in which an investor also invests in a company that it has an indirect board connection with through another firm it also invests in. In this motif of size 3, two investments (ownership links) by a particular firm are accompanied by a direct (path length 1) and an indirect (path length 2) interlocking directorate link. Of course we cannot establish causality here and determine if the interlocks lead to investments, or the other way around. The rightmost firm may very well be assigning executives to sit on both boards after they invest into them. From a corporate governance point of view, this second situation would also be highly interesting, as it all hints at the well-known monitoring function of interlocks, where a director is strategy placed to oversee a certain investment (Mizruchi 1996; Fohlin 1999). The literature on board interlock formation (Mizruchi 1996) suggests that in addition to the monitoring task, the motif in Fig. 7c may also be exemplary of the case of a trustworthy director from the perspective of the investor. Indeed, in literature it is often postulated that interlocks go together with cohesion and trust amongst the involved board members (Koenig and Gogel 1981).

Board interlocks between two investors also play a role, as Fig. 7d highlights. This motif may exemplify a coordinated investment strategy. If coordination indeed takes place between the investors, the de facto ownership concentration in the invested firm is larger than the ownership ties alone suggest. In a similar vein, Fig. 7e shows a pattern of potential hidden investment, where the sending investor holds both a direct and indirect share in the receiving firm, highlighting the opacity of corporate ownership structures (Vitali et al. 2011b; Garcia-Bernardo et al. 2017).

A final observation with respect to the motifs of size 3 is made with regards to node pairs (which could also be seen as subgraphs of size 2, for which we logically did not perform explicit enumeration). Indeed, any insight from such subgraph patterns would simply be about links and the frequency of multiplex links, not resulting in significant motifs as we fixed the coincidence of link types in the null model. However, in some of the motifs we do observe a reciprocated ownership link between a pair of nodes as part of larger ownership motifs of size 3. We acknowledge that this observation could also be made from comparing the global metric of link reciprocity (percentage of symmetric links) between the random graphs and the empirical network. Yet, it is an interesting finding as it demonstrates the existence of so-called crossholdings. A crossholding indicates a mutual investment of two firms, so a firm invests in a firm that is also its shareholder. Such structures are typically related to an institutional preference for more direct forms of economic coordination (Soskice and Hall 2001), a common phenomenon in Germany (Adams 1999).

**Size 4.** As the motif size increases, fewer of the possible subgraph patterns that may exist, actually occur in the empirical data, as can be seen in Table 3. Some of the findings that hold for size 3 motifs, such as the frequent co-occurrence of groups of firms linked through board interlocks together with ownership ties, are prevalent for size 4 as well. Indeed, 30 out of total 48 multiplex motifs of size 4 features two or more board interlocks together with a particular ownership link formation. Furthermore interesting to note is the size 4 motif in Fig. 7f, with a ratio of 2 024 and concentration of 0.351*%*. It shows how two investors have an aligned investment strategy. The division over economic sectors in Table 1 shows that 87% of the firms are in the industrial sector. In contrast, this motif’s links are between 43% industrial and 56% financial firms. It is indeed plausible that in their investment decisions, different financial firms consider similar factors when investing in industry, reflected by this motif.

**Fig. 8:**
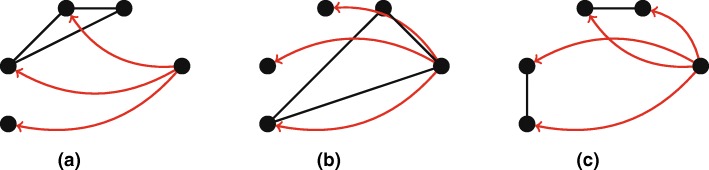
Highlighted motifs of size 5. Values of *r* and *c* denote respectively ratio and concentration cf. “Motif evaluation metrics” section. **a** Motif of size 5. *r*=285 266, *c*=0.158*%***b** Motif of size 5. *r*=2346, *c*=0.673*%***c** Motif of size 5. *r*=113 400, *c*=0.010*%*

**Size 5.** Increasing the size of motifs by yet another node, we again notice how well-connected boards appear to both attract (see Fig. 8a) and create (see Fig. 8b) more investments from firms with diverse investment strategies. From the 73 motifs of size 5, a total of 42 motifs involves an investment into or by two or more firms connected through a board interlock. Indeed, it has often been postulated in corporate governance theory that well-connected boards are more active in seeking and attracting capital elsewhere. Additionally, Fig. 8c shows a motif of size 5 with one of the highest ratio values, namely 113 400. Interestingly, it turns out that Mutual & Pension Fund firms are very frequently involved in this motif. Whereas only 0.28*%* of the firms in the data is of this type, 14% of edges in this particular motif involve such a firm. The structure represents two investments into two firms governed by the same director. Indeed, from an economic point of view it makes sense for (pension) funds not to randomly invest, but to strategically choose firms at which one knows a particular trustworthy board member from a previous investment. Interesting to note is that the size 3 version of this motif (Fig. 7c), the pattern of investment by one firm in two firms with shared directors, does not have such an over-representation of pension funds. This finding may confirm that the unique aspect of this motif is the fact that it concerns multiple diversified investments by the pension funds. Although we are not able to search for motifs larger than *k*=6, it may very well be that the diverse investment in Fig. 8c happened at a larger scale as well, with for example investments into a triangle of firms with interlocking directorates, or even three pairs of firms.

### Discussion

The first overall observation from the obtained motifs is that board interlocks and ownership links truly go hand in hand. The majority of the multiplex motifs show how well-connected firms in terms of interlocking directorates are also more involved in ownership links. This may happen in two ways: well-connected firms attract more investments, and together these firms invest more in other firms. Although we are not able to assess causality given that we do not have timestamps on the links, the observation in itself is interesting from a network analysis point of view. It is particularly interesting because the only thing fixed in the null model are the node degrees of each link type; yet at the interfirm level the co-occurrence is once again significantly present, demonstrating a higher order pattern of interlayer dependency. In a corporate network, this explicitly signals the concentration of ownership through multiple types of connections.

Apart from the motifs discussed above, a small part of the network motifs are also explainable by other means than a comparison with corporate governance practices or otherwise known corporate structures. An example is given in Fig. 7e, which displays a motif which also re-occurs in the motifs of size 4 and size 5. Upon inspection of the data, it turns out that the explanation of hidden investment given in the “Motif results” section indeed is the case, but often this patterns also appears to signal an administrative structure. An investment from parent into subsidiary and the subsidiary of that subsidiary is often done to for example separate real estate and regular business in a holding company. We acknowledge that in general, at the micro level of corporate networks, separating true business entities from administrative entities is a difficult task (see for example the discussions in Garcia-Bernardo et al. (2017); Heemskerk and Takes (2016)), and here we see how the same problem occurs at the more complex level of network motifs.

**Fig. 9:**
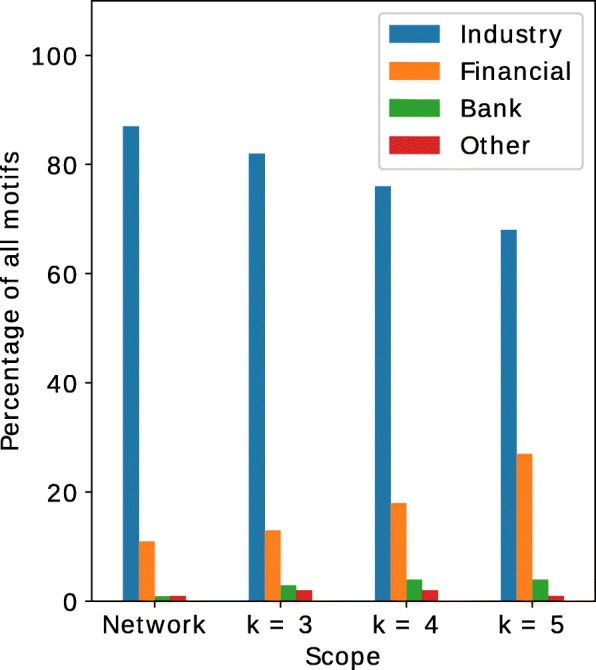
Division of firms over economic sectors, for the full network (leftmost set of bars) and motifs of different sizes (three rightmost sets of bars)

Lastly and perhaps importantly, thus far we only looked at the involvement of particular economic sectors as part of one particular motif. It could be interesting to provide a list of motifs characteristic for each node attribute value (e.g., economic sector), of each of the nodes in each discovered motif. This is however prohibitive, as extensive bookkeepping and thus an exceptionally large amount of memory would be needed to achieve this. Instead, we will look at the composition of all motifs, which provides aggregated insights per motif size. Recall that if corporate structures were to be organized according to particular motif structures without sectoral preferences, then the division of firms over economic sectors as shown in Table 1 should be the same for all motif sizes. However, as Fig. 9 shows, when the size of motifs increases, the involvement of the financial sector and banks increases at the cost of a decreasing involvement of the industry sector. This suggests that the financial sector is in general more involved in larger and more complex corporate structures. This observation essentially confirms what studies related to the financial crisis have, in a more general sense, repeatedly pointed out. There is a substantially large involvement of entities from the financial sector in the formation of more complex economic structures (Hellwig 2009; Kirkpatrick 2009).

## Conclusion

The discovery of the basic building blocks of multiplex networks is a nontrivial procedure, both methodologically and conceptually. To attain this goal, we modified an existing subgraph enumeration algorithm to handle multiplex network data. In addition, to counter the inherent interlayer dependencies of the considered multiplex corporate network, we created a null model that preserved the degree distribution of each link type, as well as the co-existence of certain types of links. A comparison of the subgraph patterns in the empirical network with those generated by the model ultimately allowed us to obtain the set of significant network motifs for our multiplex corporate network. Most notably, we demonstrated how looking at network motifs is truly able to provide new insights in the considered domain of corporate networks.

Although corporate networks had frequently been studied at a smaller scale, their meso level pattens had thus far remained undiscovered. It turns out that a number of existing theories from the field of interlocking directorates and corporate governance are nicely reflected by the obtain network motifs. Examples include ownership concentration, the monitoring function of directors, the investment diversification by pension funds and in a general sense the increased investment activity by and in firms with well-connected boards of directors. Furthermore, the obtained frequent subgraph frequencies demonstrate particular patterns of the network as a whole. Some of these patterns are characteristic for the German economy, with the appearance of so-called crossholdings as an example. Other patterns appear to be specific to certain economic sectors. Particularly noteworthy is the fact that motifs involving a company from the financial sector become more frequent as the size of the motif increases, demonstrating the role of the financial sector in creating more complex corporate structures.

Although we now have an understanding of the basic building blocks of the corporate network of Germany, in future work it could be interesting to perform a cross-country comparison, investigating if the prominent presence of particular sectors is as prevalent in other countries. The coming of age of large-scale corporate network analysis will certainly benefit from including motif analysis in the research agenda (Heemskerk et al. 2018). For example, a longitudinal analysis can reveal how the meso level building blocks of corporate networks in different countries change over time. A further investigation of the frequent patterns per attribute value could be of interest, allowing us to determine which motifs are characteristic for which economic sector. In a general sense, we hope that the data-driven insight into the organization of corporations provided by motifs at the meso level may spark new research questions and in general advance our understanding of the socio-economic system modeled by corporate networks.

Furthermore, incorporating timestamps on the edges would allow the inference of causality in the formation of particular linking structures. Although in previous work a number of economic and governance related aspects have been associated with board interlocks and ownership concentration, very few causal relationships have been confirmed. Timestamped motif detection would enable us to empirically validate on a large scale a number of theories posed in corporate governance literature about the causes and consequences of board interlocks (Mizruchi 1996). Methodologically, it would be interesting to see the effect of edge weight on the discovered motifs, posing additional challenges in the subgraph enumeration step. In corporate networks, this could be used to better distinguish between the role of majority and minority ownership on network motifs. Another interesting angle is that of anti-motifs: patterns that rarely or never occur in the empirical graph, but occur frequently in the random graphs. Finally, it could be interesting to test the algorithm on other multiplex network datasets in an attempt to unravel the universal building blocks of complex networks.
